# shRNA targeting long non-coding RNA CCAT2 controlled by tetracycline-inducible system inhibits progression of bladder cancer cells

**DOI:** 10.18632/oncotarget.8259

**Published:** 2016-03-22

**Authors:** Jianfa Li, Chengle Zhuang, Yuchen Liu, Mingwei Chen, Qing Zhou, Zhicong Chen, Anbang He, Guoping Zhao, Yinglu Guo, Hanwei Wu, Zhiming Cai, Weiren Huang

**Affiliations:** ^1^ Key Laboratory of Medical Reprogramming Technology, Shenzhen Second People's Hospital, First Affiliated Hospital of Shenzhen University, Shenzhen, China; ^2^ Shantou University Medical College, Shantou, China; ^3^ Anhui Medical University, Hefei, China; ^4^ Shanghai-MOST Key Laboratory of Health and Disease Genomics, Chinese National Human Genome Center at Shanghai, Shanghai, China; ^5^ Department of Urology, Peking University First Hospital, Institute of Urology, Peking University, National Urological Cancer Center, Beijing, China

**Keywords:** CCAT2, bladder cancer, lncRNAs, tetracycline-inducible, double shRNAs

## Abstract

Recent reports show that long non-coding RNAs (lncRNAs) are emerging as significant functional regulators in the development of tumors, including bladder cancer. Here, we found that CCAT2 was upregulated in bladder cancer tissues and cell lines. Through the statistical analyses, we also found that the high expression level of CCAT2 was positively correlated with histological grade and TNM stage of bladder cancer. Further experimental results revealed that knockdown of CCAT2 could decrease cell proliferation and migration as well as induce apoptosis in bladder cancer cells. Besides, using the post-transcriptional device of synthetic biology, we create the tetracycline-inducible double small hairpin RNAs (shRNAs) vector to control the expression level of CCAT2 which was induced by doxycycline in a dosage-dependent manner. In summary, our data indicated that CCAT2 may be an oncogene and a therapeutic target in bladder cancer. The expression of CCAT2 can be quantitatively controlled by the synthetic “tetracycline-on” switch system in bladder cancer in response to different concentrations of doxycycline to inhibit the development of bladder cancer cells.

## INTRODUCTION

Bladder cancer is one of the major common causes of global cancer mortality all over the world [1, 2]. Radiation therapy, chemotherapy and surgery are current important treatments for bladder cancer. However, the patients' 5 year survival rate is still low [3, 4]. Lacking of integrated understanding of development of bladder cancer is one of the crucial reasons. Recent studies show that some dysregulated long non-coding RNAs (lncRNAs) such as GHET1, HOTAIR, MDC1-AS may be used as therapeutic targets for patients diagnosed with bladder cancer [5–7]. It indicates that lncRNAs may act as novel indicators to cure bladder cancer.

Long non-coding RNAs (lncRNAs) participate in the development of many human diseases including cancer [8–11]. Long non-coding RNA, CCAT2, is overexpressed and promotes progression of colorectal cancer [12]. CCAT2 activates proliferation of breast tumor by regulating the Wnt signaling pathway [13]. In esophageal squamous cell carcinoma, CCAT2 is overexpressed and may be a diagnostic biomarker [14]. However, the role of CCAT2 in the development of bladder cancer is unclear and is needed to be studied.

Synthetic biology is defined as an emerging technology that combines different biological components into gene networks which confers effective biological functionalities to cells. Since synthetic biology has been successfully applied to eukaryotic cells, many researchers started to exploit new tools to enhance current technology. With the development of synthetic biology, genetic devices have been widely used to modulate signalling pathways, regulate the expression of gene and edit chromatin biochemistry [15–17]. Recently, applying synthetic biology to medicine has become a new and hot topic [15–16]. Inspired by the engineering principles of medical synthetic biology, we employed a biological device, tetracycline-inducible system, to construct the tetracycline-inducible double shRNAs targeting CCAT2. Tetracycline-inducible system is a widely used biological system and may be an available tool in the coming era of medical synthetic biology [15,18]. In addition, the tetracycline-inducible system has been developed to regulate the expression of shRNA and control the targeted gene expression through post-transcriptional modification [19]. The effective application of this biological component may provide novel approaches to treat bladder cancer.

In this study, our data showed that CCAT2 was overexpressed in bladder cancer tissues and cell lines, and promoted progression of bladder cancer cells. The synthetic “tetracycline-on” switch, was used to regulate expression of double shRNAs targeting CCAT2 in response to different contentrations of doxycycline and to suppress progression of bladder cancer cells.

## RESULTS

### CCAT2 was upregulated in bladder cancer tissues and cell lines

**Figure 1:**
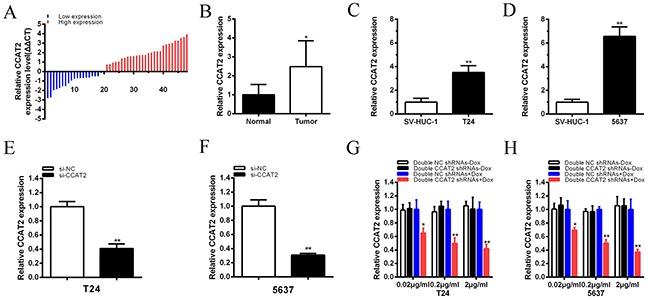
qRT-PCR method was used to detect CCAT2 mRNA level **A.** Relative concentration of CCAT2 was detected in bladder cancer tissues. **B.** Compared with matched normal tissues, the relative expression level of CCAT2 was significantly higher in bladder cancer tissues (P < 0.01). **C, D.** The relative expression level of CCAT2 was significantly upregulated in T24 (C) and 5637 (D) cells (P<0.05) compared with SV-HUC-1. **E, F.** The relative expression level of CCAT2 was inhibited by si-CCAT2 in bladder cancer T24 (E) and 5637 (F) (P < 0.01). **G, H.** Double CCAT2 shRNAs expression were induced by doxcycline and the expression of CCAT2 was down-regulated maximumly in double CCAT2 shRNAs plus 2 μg/ml doxycycline group in T24 (G) and 5637 (H) (P < 0.01) and it showed a dosage-dependent effect. Error bars suggest mean ± SD (*P < 0.05, **P < 0.01).

The relative expression level of CCAT2 was detected by performing real-time qPCR in a total of 48 bladder cancer tissues. Compared with para-cancer tissues, the CCAT2 expression was significantly upregulated in 28 cancer tissues (P<0.05) (Figure 1A and 1B). Compared with the SV-HUC-1 cell line, the expression of CCAT2 was significantly increased in T24 (P < 0.01) and 5637 (P < 0.01) (Figure 1C and 1D). As shown in Table 1, overexpression of CCAT2 was positively correlated with histological grade of bladder cancer (P = 0.014) and TNM stage (P = 0.016). However, CCAT2 expression was not associated with gender, age, tumor size and lymph node metastasis. These results showed that long non-coding CCAT2 was overexpressed in bladder cancer.

**Table 1:** Correlation between CCAT2 expression and clinicopathological characteristics of bladder cancer patients

Parameters	Group	Total	PVT1 expression		P value
			High	Low	
Gender	Male	28	17	11	0.771
	Female	20	11	9	
Age (years)	<59	13	7	6	0.750
	≥59	35	21	14	
Tumor size (cm)	<3cm	16	10	6	0.763
	≥3cm	32	18	14	
Histological grade	L	18	6	12	0.014*
	H	30	22	8	
TNM stage	0/I	12	3	9	0.016*
	II/III/IV	36	25	11	
Lymph nodes metastasis	N0	45	27	18	0.563
	N1 or above	3	1	2	

* P < 0.05 was considered significant (Chi-square test between 2 groups).

### CCAT2 promoted cell proliferation in bladder cancer cells

To investigate the functional role of CCAT2 in bladder cancer cells, qRT-PCR was used to detect the relative expression level of CCAT2 at 48h post-transfection of si-CCAT2. The relative expression level of CCAT2 in T24 and 5637 cells were significantly down-regulated by si-CCAT2 (P<0.01 in two cell lines) (Figure 1E and 1F).

To quantitatively inhibit CCAT2 expression, we also construct a tetracycline-inducible CCAT2 shRNA. Bladder cancer cells were maintained in 6-well plates and transfected with plasmids expressing either the corresponding tetracycline-inducible CCAT2 shRNA or the negative control. qRT-PCR was employed to detect the relative expression level of CCAT2. The CCAT2 shRNA induced by doxycycline could significantly suppress the relative expression level of CCAT2 in T24 and 5637 cells when added with different concentrations of doxycycline (P < 0.01 in two cell lines) (Figures 1G and 1H). When 2 μg/ml doxycycline was added to cells transfected with CCAT2 shRNA plasmids, the expression level of CCAT2 in tetracycline-inducible CCAT2 shRNA group was decreased by 58% in T24 (P < 0.01) and decreased by 63% in 5637 (P < 0.01). And it showed that doxycycline induced the expression of CCAT2 shRNA to inhibit the expression of CCAT2 in a dosage-dependent manner. Since 2 μg/ml of doxycycline inhibited the expression of CCAT2 maximally, we chose this concentration for further study.

**Figure 2:**
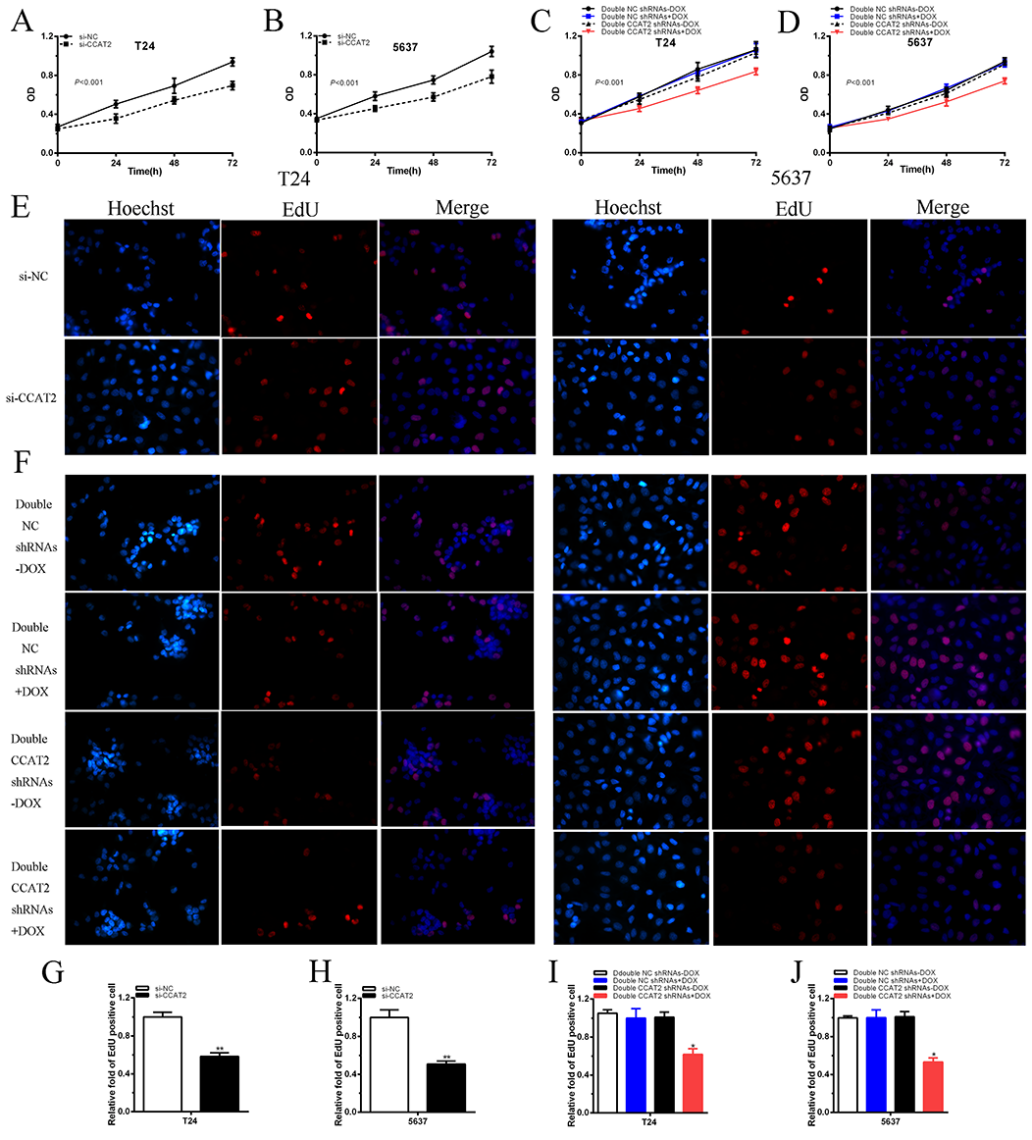
Cell growth was suppressed after transfection of special RNA or tetracycline-inducible shRNA vectors **A, B.** After transfection of si-CCAT2, cell proliferation was suppressed obviously in T24 (A) and 5637 (B) (P<0.001). **C, D.** After transfection of double CCAT2 shRNAs plus 2 ug/ml doxycycline, cell proliferation was inhibited significantly in T24 (C) and 5637 (D) (P<0.001). **E, F.** Representative images of EdU incorporation assay in bladder cancer T24 and 5637. After transfection of si-CCAT2, EdU positive cells were decreased in T24 and 5637 (E). EdU positive cells were reduced after transfection of double CCAT2 shRNAs plus doxycycline in T24 and 5637 (F). **G, H, I, J.** EdU incorporation rate was suggested as ratio of EdU positive cells relative to Hoechst 33342 positive cells. Error bars indicate mean ± SD (*P < 0.05, **P < 0.01).

CCK-8 assay results suggested that si-CCAT2 significantly decreased cell proliferation in bladder cancer cells (p < 0.001 in two cell lines) (Figure 2A and 2B). CCK8 was also performed to detect whether cell proliferation was decreased by tetracycline-inducible CCAT2 shRNA. The results showed that, compared with the negative control, tetracycline-inducible CCAT2 shRNA significantly suppressed proliferation when added with 2 ug/ml doxycycline in bladder cancer T24 and 5637 cells (P < 0.001 in two cell lines) (Figure 2C and 2D).

Then EdU assay was carried out to further detect function of CCAT2 in promoting cell proliferation. As shown in Figure 2E, compared with si-NC group, the rate of EdU positive T24 or 5637 cells were significantly decreased in si-CCAT2 group. As shown in Figure 2F, less EdU positive T24 or 5637 cells were observed in group transfected with 2 ug/ml tetracycline-inducible CCAT2 shRNA.

The quantitative results proved that the number of EdU positive cells in si-CCAT2 group was significantly reduced in T24 (P < 0.01) and 5637 (P < 0.01) (Figure 2G and 2H). The quantitative results also suggested that, compared with the negative control group, the rate of EdU positive cells in group transfected with 2 ug/ml tetracycline-inducible CCAT2 shRNA was significantly decreased in T24 (P < 0.01) and 5637 (P < 0.01) (Figures 2I and 2J).

These data indicated that CCAT2 may promote cell proliferation in bladder cancer.

### CCAT2 promoted cell migration in bladder cancer cells

**Figure 3:**
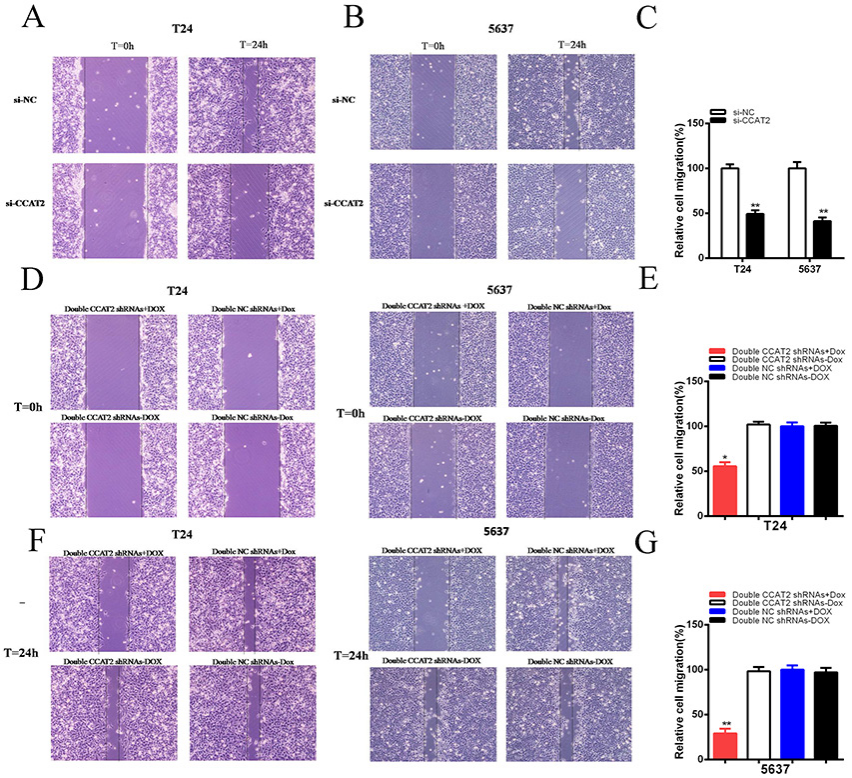
Cell migration was suppressed after transfection of si-CCAT2 or double CCAT2 shRNAs vectors **A, B.** Representative images of cell migration were shown after transfection of specific RNA in bladder cancer. Cell migration was inhibited obviously in T24 (A) and 5637 (B). **C.** Relative cell migration was suppressed significantly after transfection of si-CCAT2 in T24 and 5637. **D, E, F, G.** Representative images of cell migration were shown after transfection of shRNAs vector in T24 and 5637 (D, F). Relative cell migration was inhibited significantly after transfecting double CCAT2 shRNAs plus doxycycline into T24 (E) and 5637 (G). Error bars show mean ± SD (*P < 0.05, **P < 0.01).

To investigate whether CCAT2 promotes cell migration of bladder cancer cells, cells were transfected with si-NC or si-CCAT2 in 6-well plates. As shown in Figures 3A, 3B and 3C, compared with the negative control, cell migration ability was obviously inhibited in si-CCAT2 (P<0.01 in two cell lines). As shown in Figures 3D, 3E, 3F and 3G, compared with the negative control, cell migration ability was obviously suppressed in bladder cancer cells transfected with tetracycline-inducible CCAT2 shRNA group (P<0.05 in T24 and P<0.01 in 5637). These results indicated that CCAT2 should increase cell migration in bladder cancer.

### CCAT2 inhibited cell apoptosis in bladder cancer cells

**Figure 4:**
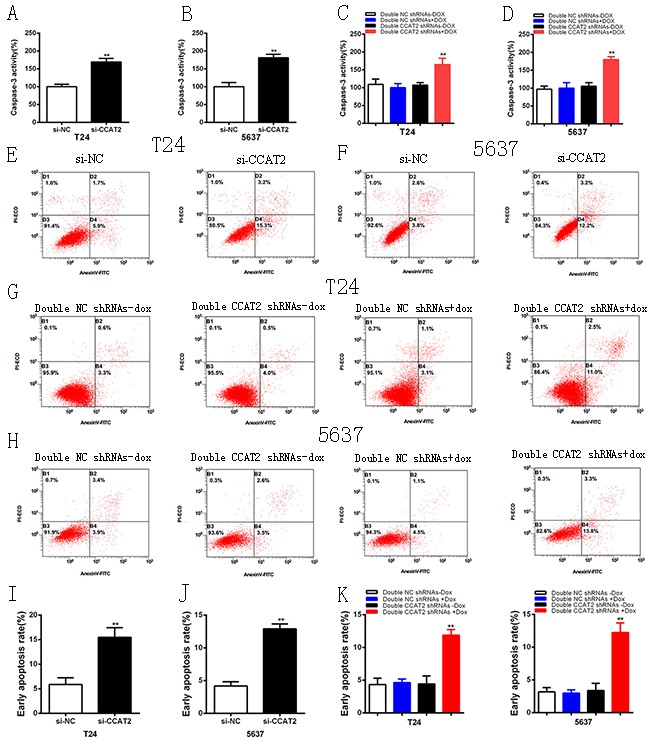
Apoptosis was induced after transfection of special RNA or double tetracycline-inducible shRNAs vectors using ELISA and flow cytometry assay **A, B.** The activity of caspase-3 was increased significantly after transfection of si-CCAT2 in T24 (A) and 5637 (B). **C, D.** The activity of caspase-3 was enhanced significantly after transfection of tetracycline inducible double shRNAs vectors in T24 (C) and 5637 (D). **E, F, I, J.** Compared with si-NC group, more apoptotic cells were measured in si-CCAT2 group in T24 (E, I) and 5637 (F, J). **G, H, K, L.** More apoptotic cells were detected in double CCAT2 shRNAs plus doxycycline group compared with the corresponding negative control vectors in T24 (G, K) (P < 0.01) and 5637 (H, L) (P < 0.01). Error bars suggest mean ± SD (*P < 0.05, **P < 0.01).

To investigate whether CCAT2 suppresses apoptosis of bladder cancer cells, cells were transfected with si-CCAT2 or si-NC and the caspase 3 enzyme-linked immunosorbent assay (ELISA) and flow cytometry assay were used to detect cell apoptosis. As shown in Figure 4A and 4B, compared with cells transfected with si-NC, the activities of caspase 3 (P < 0.01 in two cell lines) were significantly increased. Also shown in Figure 4C and 4D, Compared with the negative control, the relative activity of caspase-3 was significantly increased in double CCAT2 shRNAs induced by 2 ug/ml doxycycline (P < 0.01 in two cell lines). Flow cytometry assay data also showed that the early apoptosis ratio (P < 0.01 in two cell lines) was significantly increased in cells transfected with the si-CCAT2 (Figure 4E and 4F). As shown in Figure 4G and 4H, compared with the negative control group, the early apoptosis ratio was significantly increased in tetracycline-inducible CCAT2 shRNA group (P < 0.01 in two cell lines). The quantitative results also confirmed these results (Figures 4I, 4J, 4K and 4L). The data suggested that CCAT2 inhibited cell apoptosis of bladder cancer cells.

## DISCUSSION

Previous studies show that lncRNAs participate in gene regulation and promote our understanding of the mechanism of disease development, including cancers [20, 21]. CCAT2 is located in chromosome 8q24 [12]. CCAT2 is overexpressed and increases cell proliferation through the Wnt signaling [13, 22]. In gastric cancer, CCAT2 was upregulated and used as a potential indicator of prognosis [23]. Besides, CCAT2 promotes invasion of non-small cell lung cancer [24]. However, the relationship between CCAT2 and bladder cancer is unclear.

It is the first study to investigate the functions of CCAT2 in bladder cancer. In this study, data showed that the expression of non-coding RNA CCAT2 was upregulated in bladder cancer tissues and cell lines. High expression of CCAT2 was also positively associated with TMN stage and histological grade of bladder cancer. To further study the biological behaviors of CCAT2 in bladder cancer, we employed the CCK8, EdU assays, cell migration assay and cell apoptosis assays to detect cell growth, migration and apoptosis in bladder cancer cells through knock down of CCAT2. After silencing CCAT2, cell growth and cell migration were inhibited and cell apoptosis was increased in bladder cancer cells. These results indicated that CCAT2 promoted the progression of bladder cancer.

Synthetic biology is now emerging as a novel field in gene therapy. Through the biological devices, such as transcriptional switches, post-transcriptional switches, promoters and logic gates, complex synthetic gene networks and genetic circuits were conducted to mediate signal transduction, genome editing and chromatin modification [15–17]. These devices are employed in the emerging era of medical synthetic biology. What's more, recent studies show that we can use medical synthetic biology to treat bladder cancer. Through the CRISPR-Cas9 system, a new device of synthetic biology, Liu et al [25] constructed modular AND gate circuits to inhibit bladder cancer cell growth. Tetracycline-inducible system, another important biological component, was widely used in gene regulation. Our group has employed the tetracycline-inducible system to control the expression of PVT1 [26]. In addition, the Tetracycline-inducible system can effectively regulate the tet-inducible artificial microRNAs to silence the oncogene in bladder cancer [27].

In this study, we reported the construction of tet-inducible system of CCAT2 and detected its anti-cancer effects in bladder cancer. Our data showed that the tet-inducible system could control two identical shRNAs targeting CCAT2 in a dosage-dependent manner and effectively inhibited the development of bladder cancer cells. These observations also suggested that the anticancer efficiency could be further increased if more different shRNAs and more cancer-related genes were controlled.

In conclusion, CCAT2 promotes progression of bladder cancer cells. Furthermore, we use the one of the synthetic devices, tetracycline-inducible switch, to control the expression of shRNA targeting CCAT2, thus providing a novel method in medical synthetic biology. Utilization of this synthetic biology device to construct tet-inducible shRNA is a simple and cheap approach which has presented a good prospect to treat cancer. Future therapeutic schemes about gene therapy and medical synthetic biology can take this research into consideration. At last, CCAT2 may become a novel molecular target for bladder cancer treatment and this device may be widely used in the cancer therapy in the future.

## MATERIALS AND METHODS

### Cell culture

SV-HUC-1 cells and human bladder cancer cells (T24, 5637) were purchased from the Institute of Cell Research, Chinese Academic of Sciences, Shanghai, China. The SV-HUC-1 cells were cultured in F12K medium (Invirtogen, Carlsbad, CA, USA) plus 1% antibiotics (100U/ml penicillin and100 μg/ml streptomycin sulfates) and 10% fetal bovine serum (FBS) at 37°C with an atmosphere of 5% CO_2_ in incubator. T24 and 5637 were cultured as suggested by American Type Culture Collection (ATCC, Manassas, VA).

### Patient samples

In this research, 48 patients diagnosed with bladder cancer were included. Matched para-cancer tissues and bladder cancer tissues were cut and then snap-frozen in liquid nitrogen immediately. Written formal approval was also gotten from all the patients. This research was approved by the Institutional Review Board of Shenzhen Second People's Hospital.

### Creation of synthetic tetracycline-inducible shRNA vector targeting CCAT2

Tetracycline-inducible double shRNA targeting CCAT2 and the negative control were inserted into tetracycline-inducible plasmid vectors psi-LVRInU6TGP that was brought from FulenGen firm, Guangzhou, China. The tetracycline-inducible double shRNAs were generated with the primary sequence “TTAACCTCTTCCTATCTCA”.

### Transfection of cell lines

Bladder cancer cells were transiently transfected with specific siRNA oligonucleotides and tetracycline-inducible double shRNAs vectors using Lipofectamine 2000 Transfection Reagent (Invitrogen, Carlsbad, CA, USA) according to the manufacturer's protocol. We chose the sequence, ‘TTAACCTCTTCCTATCTCA’ for further study [12]. Non-specific siRNA (si-NC) and si-CCAT2 were purchased from GenePharma, Suzhou, China. Plasmid Midiprep kits (Promega, Madison, USA) were utilized to get the plasmid vectors (CCAT2 shRNA, NC shRNA) before transfection.

### RNA extraction and qRT-PCR analysis

Total RNAs from tissues or cells after transfection were extracted using the TRIzol reagent (Invitrogen, Grand Island, NY, USA) according to the manufacturer's instructions. PrimeScript RT Reagent Kit with gDNA Eraser (Takara, Japan) was used to transform RNA to cDNA. The mRNA expression levels of CCAT2 were measured by using SYBR® Premix Ex TaqTM (TaKaRa, Japan) according to the user's manuals on the Roche lightcycler 480 Real-Time PCR System. GAPDH was utilized as the control to normalize the data. The primer sequences were shown: CCAT2 primers [22] forward: 5′-CCCTGGTCAAATTGCTTAACCT-3', reverse: 5′-TTATTCGTCCCTCTGTTTTATGGAT-3'; GAPDH primers forward: 5′-CGCTCTCTGCTCCTCCTGTTC-3', reverse: 5′-ATCCGTTGACTCCGACCTTCAC-3'. The comparative ΔCt method was used to analyze the results by calculating the relative amount of CCAT2. All experiments were carried out at least three repetitions.

### Cell proliferation assays

Cell Counting Kit-8, CCK-8 (Beyotime Institute of Biotechnology, Shanghai, China) and 5-ethynyl-20-deoxyuridine (EdU) assay kit (Ribobio, Guangzhou, China), respectively, were used to measure cell proliferation according to the manufacturer's instructions. 4×10^3^ cells per well were seeded in a 96-well plate and pre-incubated for 24 hours, and then transiently transfected with siRNAs or plasmids. At 24, 48 or 72 h post-transfection, 10 μl CCK-8 was added to each well and cultured for 1 h. Absorbance at a wavelength of 450 nm was measured by an ELISA microplate reader (Bio-Rad, Hercules, CA, USA). EdU incorporation assay was carried out according to previous studies [26, 27]. The experiments were performed in triplicate.

### Cell migration assay

Cell migration was measured by scratch assay. Cells were seeded in 6-well plates and cultured for 24 hours before transfection. The cells were transfected with specific siRNA oligonucleotides or vectors. Clear lines in the wells were generated using a sterile 200 μl pipette tip. A digital camera system was used to take pictures from each well quickly. 24 hours later, pictures were taken again. Migration distance was measured at the time of 0h and 24h. Each test was carried out at least three times.

### Cell apoptosis assays

ELISA assay and flow cytometry assay were used to detect cell apoptosis. Bladder cancer cells were transiently transfected with siRNAs or plasmids in 12-well plates. After 48 hours, the caspase 3 enzyme-linked immunosorbent assay (ELISA) assay kit (Hcusabio, Wuhan, China) was used to detect the activity of caspase 3 according to the manufacturer's protocol. For flow cytometry assay, cells were cultivated in 6-well plates before transfection. 48 hours after transfection, cells were collected and washed with PBS. The annexin V-fluorescein isothiocyanate (FITC)/PI detection kit (Invitrogen, Carlsbad, CA, USA) was used to stain cells with Annexin V-FITC (AV, 5μl) and propidium iodide (PI, 3μl) according to the manufacturer's manuals. Flow cytometry (EPICS, Xl-4, Beckman, CA, USA) was employed to detect the percentage of apoptotic cells. In the graphs, four quadrants were shown and cells were discriminated into dead cells (upper left), living cells (lower left), early apoptotic cells (lower right) and late apoptotic cells (upper right). The ratio of early apoptotic cells was regarded as an observation index to compare the experimental group and negative control group. All experiments were repeated at least three times.

### Statistical analysis

All data were presented as mean ± standard deviation (SD) from three independent experiments. All statistical analyses were executed by using SPSS 21.0 software (IBM, Chicago, IL, USA). CCAT2 expression difference between bladder cancer tissues and para-cancer tissues was analyzed using paired samples' *t* test. Cell proliferation data (CCK-8 assay) were analyzed by ANOVA and independent samples' *t* test was used to analyze other data. A two-tailed value of P < 0.05 was considered statistically significant.
